# Character-based DNA barcoding allows discrimination of genera, species and populations in Odonata

**DOI:** 10.1098/rspb.2007.1290

**Published:** 2007-11-08

**Authors:** J Rach, R DeSalle, I.N Sarkar, B Schierwater, H Hadrys

**Affiliations:** 1 ITZ, Ecology and EvolutionTiHo Hannover, Bünteweg 17d, 30559 Hannover, Germany; 2 Division of Invertebrate Zoology, American Museum of Natural HistoryNew York, NY 10024, USA; 3 MBLWHOI Library, Marine Biological LaboratoryWoods Hole, MA 02543, USA; 4 Department of Ecology and Evolutionary Biology, Yale UniversityNew Haven, CT 06520-8104, USA

**Keywords:** character-based DNA barcoding, characteristic attributes organisation system, Odonata, ND1, conservation genetics, biodiversity

## Abstract

DNA barcoding has become a promising means for identifying organisms of all life stages. Currently, phenetic approaches and tree-building methods have been used to define species boundaries and discover ‘cryptic species’. However, a universal threshold of genetic distance values to distinguish taxonomic groups cannot be determined. As an alternative, DNA barcoding approaches can be ‘character based’, whereby species are identified through the presence or absence of discrete nucleotide substitutions (character states) within a DNA sequence. We demonstrate the potential of character-based DNA barcodes by analysing 833 odonate specimens from 103 localities belonging to 64 species. A total of 54 species and 22 genera could be discriminated reliably through unique combinations of character states within only one mitochondrial gene region (NADH dehydrogenase 1). Character-based DNA barcodes were further successfully established at a population level discriminating seven population-specific entities out of a total of 19 populations belonging to three species. Thus, for the first time, DNA barcodes have been found to identify entities below the species level that may constitute separate conservation units or even species units. Our findings suggest that character-based DNA barcoding can be a rapid and reliable means for (i) the assignment of unknown specimens to a taxonomic group, (ii) the exploration of diagnosability of conservation units, and (iii) complementing taxonomic identification systems.

## 1. Introduction

To reliably identify and monitor biodiversity in the field and pinpoint more efficiently small but important areas of conservation is still a major challenge. In this context, DNA barcoding has gained great attention as a universal means for the identification of organisms. In several animal groups, specimens have been assigned to species, cryptic species have been discovered and clusters within species have been detected by short DNA sequences of a standardized gene region (Hebert *et al*. 2003*a*; Ward *et al*. 2005; Gomez *et al*. 2007; Smith *et al*. 2006).

Not all potential uses that have been suggested for DNA barcodes are considered valid. Among these uses are two that are often confused—species identification and species discovery (see Desalle 2006; Rubinoff 2006*b*). Species identification is unequivocally a valid use of DNA barcoding. Here, DNA sequences are used as markers for *a priori* established species. In the context of species identification, DNA barcoding does not rely on a species concept. In fact, species identification using DNA barcoding is consistent with any species concept that a taxonomist uses to establish a named species. Species discovery on the other hand is a much more complex matter. Species discovery is really the job of taxonomy and hence cannot solely use DNA barcodes as the arbiter of the discovery of new species (DeSalle *et al*. 2005; Desalle 2006). Species discovery requires a species concept and a corroboration system (DeSalle *et al*. 2005) and in this sense no single source of data—be it DNA, morphology, ecology, reproductive isolation or behaviour—can by itself be used to discover species. However, we point out that DNA sequences can be used to ‘flag’ potential new species units. In the context of diagnosability, DNA diagnostics discovered at the population level or in situations where crypticism might occur can be used to propose (flag) new hypotheses of species existence, which then need to be corroborated using an integrated taxonomic approach (Rubinoff 2006*a*,*b*), or a well-established species discovery approach, both of which require a species concept.

It has become apparent that the currently used genetic distance approaches for using (‘reading’) DNA barcodes have strong limitations, particularly when it comes to defining species boundaries (Witt *et al*. 2006). Although some studies have been successful in defining DNA barcodes by means of genetic distance thresholds, for example, in butterflies and crustaceans (Hebert *et al*. 2004*b*; Lefebure *et al*. 2006), distance threshold boundaries seem to be ill suited as a general means for species identification (Rubinoff 2006*b*; Rubinoff *et al*. 2006). One reason is that mtDNA rates of evolution vary substantially between and within species and between different groups of species resulting in broad overlaps of intra- and interspecific distances (Kipling & Rubinoff 2004; Rubinoff 2006*b*; Rubinoff *et al*. 2006). These overlaps may hinder the accurate assignment of query sequences with distance-based methods, especially in cases of insufficient taxon sampling (Meyer & Paulay 2005; Wiemers & Fiedler 2007). An alternative to existing phenetic approaches is the character-based DNA barcoding (DeSalle *et al*. 2005). In the present scenario, previously established taxonomic groups are identified through the presence of diagnostic characters or combination of characters within short stretches of DNA sequences. In this sense, character-based DNA barcoding is consistent with and can serve as a complement to the approaches of traditional morphological identification systems. The main difference is that the number of diagnostic characters in a molecular approach can cum grano salis be unlimited.

The characteristic attribute organization system (CAOS) is based on the fundamental concept that members of a given taxonomic group share attributes (e.g. polymorphisms) that are absent from comparable groups (Sarkar *et al*. 2002*b*). The CAOS algorithm thus identifies character-based diagnostics, here termed ‘characteristic attributes’ (CAs), for every clade at each branching node within a guide tree that is first produced from a given dataset. The resulting diagnostics can then be used for subsequent classification of new data into the taxonomic groupings represented by the guide tree. The guide tree is used by CAOS only as a means to identify diagnostic characters; it does not necessarily represent putative phylogenetic relationships. Thus, the guide tree can be generated using any number of tree-building methods (Sarkar *et al*. 2002*b*).

CAs are diagnostic character states (genes, amino acids, base pairs or even morphological, ecological or behavioural attributes) which are found only in one clade but not in an alternate group that descends from the same node. CAs are then divided into two major groups: (i) *pure* CAs are shared by all members of the clade and are absent from the other clades, while (ii) *private* CAs are shared only by some members of a clade but are absent from the other clades. Both *pure* and *private* CAs can either be *simple* CAs, which are confined to a single nucleotide position, or *compound* CAs which are combined states at multiple nucleotide positions. The latter, which are not characteristic in and of themselves, are diagnostic when this combination occurs only in one of the clades at a given node (see DeSalle *et al*. 2005). In this study, we took the most conservative approach by only considering *simple pure* (sPu) and *simple private* (sPr) CAs that are shared in at least 80% of all members in a given taxonomic unit. After CAs have been identified for a given taxonomic group, they can be used as diagnostic standard—a ‘character-based DNA barcode’—for this particular group.

While current barcoding studies have primarily focused on a single marker gene—the mitochondrial cytochrome *c* oxidase I (*CO1*) gene—as a source for identifying diagnostic barcodes (e.g. Hebert *et al*. 2003*b*; Armstrong & Ball 2005; Blaxter *et al*. 2005; Janzen *et al*. 2005), other markers have been suggested as equally well suited (e.g. Markmann & Tautz 2005; Monaghan *et al*. 2005; Savolainen *et al*. 2005). In this study, the mitochondrial *ND1* gene region (NADH dehydrogenase 1) will be explored for finding character-based DNA barcodes specific for taxonomic units within the insect order Odonata (dragonflies and damselflies). *ND1* has been successfully applied to phylogenetic and population genetic studies in Odonates before and seems to be well suited as an alternative or complement to *CO1* (Hadrys *et al*. 2006; Dijkstra *et al*. 2007; Groeneveld *et al*. 2007).

Odonates provide an ideal platform for exploring the potential of character-based DNA barcoding. They represent a species rich, yet tractable (approx. 5600 described species), insect order. Their different levels of habitat specificity and complex aquatic/terrestrial life cycles make them prominent surrogates for evaluating all types of freshwater ecosystems worldwide. While the imagos of the vast majority of species are readily identified by morphological, behavioural and life history traits, discrimination of the crucial larval stages still remains a major obstacle (Corbet 1999). This is unfortunate since fast and reliable identification of the larval biodiversity is instrumental in monitoring freshwater quality. A second challenge that slowly emerges is the discovery of extraordinary cryptic genetic diversity below the species level (Hadrys *et al*. 2005; Watts *et al*. 2007).

Below, we introduce the character-based DNA barcoding technique to detect diagnostic characters within the mitochondrial *ND1* gene region in a large dataset that allows for unambiguous identification of genera, species and diagnostic entities (conservation units, CUs or synonymously used ‘evolutionary significant units’; see Vogler & Desalle 1994) below the species level in dragonflies.

## 2. Material and methods

### (a) Sample collection

Tissue samples of 833 specimens from 103 localities representing 25 genera and 64 species (including several closely related species groups) were collected mostly by non-invasive sampling (Hadrys *et al*. 2005) and stored in 70 or 98% ethanol prior to DNA extraction. Table S1*a*,*b* (see electronic supplementary material) provides an overview of all species, sample sizes and localities included in this study.

### (b) DNA extraction, *ND1* amplification and sequencing

DNA was extracted according to a modified standard protocol (Hadrys *et al*. 1992). The tissue samples were freeze-dried with liquid nitrogen for better homogenization.

PCR was performed with the specific primers P850 (fw) 5′ TTC AAA CCG GTG TAA GCC AGG 3′ and P851 (rev) 5′ TAG AAT TAG AAG ATC AAC CAG 3′ amplifying an approximately 580 bp long fragment of the mitochondrial genome, which includes fragments of the 16S rRNA, the intervening tRNA^Leu^ region and the *ND1* gene region. The 25 μl reaction mixes contained: 1× amplification buffer (20 mM Tris–HCl, pH 8.4; 50 mM KCl; Invitrogen), 2.5 mM MgCl_2_, 0.1 mM dNTPs, 7.5 pM each primer and 0.5 U Taq DNA polymerase (Invitrogen). The PCR thermal regime for amplification was: 2 min at 95°C, followed by 30 cycles of 30 s at 95°C, 30 s at 49°C and 1 min at 72°C and a final elongation of 6 min at 72°C. Sequencing reactions were carried out bidirectionally on a MegaBACE 500 sequencer using the DYEnamic ET Dye Terminator Cycle Sequencing Kit (Amersham Bioscience). Forward and reverse strands were assembled and edited using SeqManII (v. 5.03; DNASTAR, Inc.) and consensus sequences were aligned using Muscle (Edgar 2004). All 833 sequences were shortened to 480 bp of an unambiguously alignable core region. Reference sequences for all 64 species were deposited into Genbank under the accession numbers EU183234–EU183297.

### (c) DNA barcoding at different levels: a taxonomy of identifiers

#### (i) Species

DNA barcodes at the species level are most appropriately used in the species identification process, but cannot be used alone in the species discovery process (but see Vogler & Monaghan (2007) for an alternative view of species discovery and DNA barcodes). For the latter, barcodes should be combined with other information in an integrated taxonomy approach (Rubinoff 2006*a*,*b*). Since species identification requires that species be defined *a priori* to the determination of DNA identifiers, DNA barcoding works with any species concept. In this paper, the determination of diagnostic DNA barcodes for the various odonate species that have existing taxonomy is accomplished by the CAOS approach described below.

#### (ii) Higher taxonomic levels

Higher taxonomic levels may also be ‘identified’ with DNA barcodes just like species can be identified. If the higher level is proscribed *a priori*, then ‘identification’ of that higher category with DNA barcodes is a simple mechanical matter of finding data that diagnose these higher categories. The discovery process for higher categories is more difficult to define since there are such broad criteria for these higher categories. One approach is to use phylogenetic analysis to establish the higher categories based on monophyly. In these phylogenetic cases, the simplest and most reliable identifiers for the higher categories are the synapomorphies for the higher category that have CI=1.0. Complex identifiers can also be used to diagnose these higher categories as we describe in the CAOS procedure below.

#### (iii) Lower categories

Lower categories such as subspecies, semi-species and populations pose special problems for DNA barcoding. These lower categories, like species and higher categories, are based on some *a priori* notion of a geographical, genetic, ecological or other criteria. In some respects, they are hypotheses of potential species existence. In this context, a population or a subspecies defined *a priori* based on some criteria other than DNA can be tested as potential species using DNA (DeSalle *et al*. 2005). In fact, if a unique DNA barcode is found for a population or subspecies, then we suggest that this evidence should be useful for flagging those populations or subspecies for further taxonomic investigation. We use the CAOS procedure described below to examine predetermined populations of odonates as potential species. We point out that the existence of DNA diagnostics for these population-level analyses require further taxonomic investigation before these newly found diagnosable populations are called new species.

### (d) Application of CAOS as a character-based barcode identifier

In order to accommodate DNA barcoding studies, we slightly modified the original CAOS algorithm (Sarkar *et al*. 2002*a*,*b*). CAOS is implemented with a series of programs (P-Gnome; http://www.genomecurator.org/CAOS/P-Gnome/PGnomeindex.html). The steps involved in identification of diagnostic characters are listed below.

- The first step in the CAOS algorithm is to generate a guide tree (Sarkar *et al*. 2002*a*). Sequence divergences among individual *ND1* sequences were estimated by using the Kimura-2-Parameter (K2P) distance model and graphically displayed in a neighbour-joining (NJ) tree (see figure S1 electronic supplement material) using PAUP (v. 4.0b10; Swofford 2002). The tree and corresponding NEXUS file were entered and saved as one file in MacClade 4 (v. 4.06; Maddison & Maddison 2000).
- Next, P-Gnome is used to search for CAs at all nodes in the guide tree generated. The CA search is performed node-by-node, beginning at the root node and continuing until the highest resolution is achieved (Sarkar *et al*. 2002*b*). P-Gnome discovers all CAs regardless of their rank (Sarkar *et al*. 2002*a*). Even private CAs that occur in only one or a few samples within a group are listed as putative diagnostic characters.
- In order to select for unambiguous diagnostic characters, we developed a program called ‘diagViewer’ and incorporated it into the CAOS/P-Gnome package. The diagViewer application removes CAs that are not shared by at least 80% of all members within a group. All pure CAs (shared by all members of one group) and private CAs (≥ 80%) are listed in the ‘diagView_attributes’ output file created by the P-Gnome application.
- A number is used to identify each node and the group at that node. The numbers of nodes and groups as well as the samples assigned to each group are listed in the ‘CAOS-group file’. Nodes that we deemed relevant for this part of the study were selected by eye and subsequently, all non-relevant nodes were sorted out by a script, called the BarcodeFilter. Nodes were considered non-relevant when no further resolution at the respective taxonomic level was achieved (e.g. nodes within species cluster are not relevant when defining species barcodes). Our final file shows all the pure CAs and the private CAs ≥ 80% for each group at the relevant nodes.
- The file from step (iv) was subsequently converted into a tab-delimited file importable to Microsoft Excel by a script, called the ‘BarcodeMaker’. Finally, a few nucleotide positions showing the highest number of CAs within the *ND1* fragment were selected. The character states (nucleotides) at these positions were listed for each species and the combination of character states were compared. In cases where a shared combination of nucleotides was observed for two or more species and no other CAs could be found, additional analyses were performed. The relevant sequences were selected from the alignment and analysed separately. Nucleotide positions at which pure CAs and private CAs ≥ 80% were found for these particular species were also included in the final table.

### (e) Application of CAOS to identify diagnostic characters for odonate genera

Some of the analysed odonate genera did not form monophyletic groups in the NJ tree. To allow a full CA search with CAOS between genera, all samples belonging to the same genus were integrated into one group. For this, the NJ tree was edited manually in MacClade (data not shown). Clades containing closely related genera descended from the same node within the tree. Nucleotide positions with the highest number of CAs were selected and the character states at these positions were listed for each genus.

### (f) Application of CAOS to identify diagnostic characters for odonate populations

For exploring the potential of the CAOS technique to identify character-based DNA barcodes of single populations, we selected a data subset of the family Coenagrionidae from the original alignment. The alignment contained *ND1* sequences of 133 specimens belonging to 14 species of Coenagrionidae. A second NJ tree based on K2P distances was generated and subsequently analysed with the methods described above (figure 1).

For this part of the study, nodes *within* species clusters of the NJ tree were considered. By means of the CAOS-group file, geographical clusters were discovered. Pure CAs found at nodes from which these particular groups descended were selected from the ‘diagView_attributes file’ and listed in a table.

## 3. Results

### (a) Character-based DNA barcodes for odonate species

Table 1 shows the character states at 23 nucleotide positions of the *ND1* gene region for 14 species of the family Coenagrionidae. The characterstates at these nucleotide positions for all 64 species are shown in table S2 (electronic supplementary material). The particular nucleotide positions were chosen due to the high number of CAs at the important nodes or because of the presence of CAs for groups with highly similar sequences. Grey-shaded cells indicate that at least three different nucleotides occurred within a species at this particular nucleotide position. These positions were considered as ‘non-significant’.

Out of 64 species, 54 immediately revealed a unique combination of character states at 23 nucleotide positions with at least three CAs for each species. For 10 species (all closely related sister taxa) less than three diagnostic characters were identified, including the two subspecies of *Aeshna ellioti*, *Aeshna ellioti usambarica* and *Aeshna ellioti ellioti*. The same applies to sequences of *Pseudagrion niloticum* (six individuals) and *Pseudagrion acaciae* (four individuals) as well as *Calopteryx virgo* (five individuals) and *Calopteryx splendens* (20 individuals). For the third species of the genus Calopteryx, *Calopteryx haemorrhoidalis*, one nucleotide position (234) was found at which all analysed samples differ from the two sister species, but five more diagnostics (nucleotide positions 201, 228, 318, 367 and 429) were identified at which 20 out of the 21 specimens of *C. haemorrhoidalis* differ from *C. virgo* and *C. splendens*. For *Aeshna grandis* and *Aeshna cyanea*, only one diagnostic character distinguishing the two sequences was detected within the *ND1* fragment (nucleotide position 367; C→T). The *ND1* sequence of *Anax parthenope* was also similar to the 88 sequences of the sister species *Anax imperator*, but separate analyses of the two species revealed two diagnostic characters at nucleotide positions 429 (G→A) and 444 (C→T; table S2, electronic supplementary material).

### (b) Character-based DNA barcodes for odonate genera

In table S3 (electronic supplementary material), the character states for 25 odonate genera at 30 nucleotide positions of the *ND1* gene region are shown. Dashed cells indicate non-significant positions at which at least three different nucleotides occurred within a genus. Unique combinations of at least three diagnostic character states were found for 21 out of 25 genera. Within the family Aeshnidae (18 species analysed), four out of six genera showed no or only one diagnostic character (marked in red; table S2). For the genus *Gynacantha*, one diagnostic character was found at nucleotide position 312 (T→A). For *Anaciaeshna*, also one diagnostic character was detected to discriminate the samples of this genus from the three other aeshnid genera (nucleotide position 357; G/C→A). No diagnostic characters were found for the genera *Aeshna* and *Anax*.

### (c) Character-based DNA barcodes identifies entities at the population level

*ND1* sequences of members of 14 species of the family Coenagrionidae were selected from our original dataset, including 13 species of the genus *Pseudagrion* and one species of the genus *Teinobasis*. The NJ guide tree for CAOS analysis of this subset contained sequences of 133 specimens (figure 1). The majority of specimens (98) belong to three *Pseudagrion* species collected at 18 different localities (table S4), providing us with the opportunity to test the power of character-based DNA barcoding at the population level. The three species, *Pseudagrion bicoerulans*, *Pseudagrion kersteni* and *Pseudagrion massaicum* formed defined clades in the guide tree (figure 1; framed in grey). Furthermore, within the species clades, geographical clusters could be detected (figure 1; highlighted by different colour codes).

We have already shown above that the discrimination of 12 species of Coenagrionidae could be achieved through character-based DNA barcoding. The only exceptions were the two species *P. acaciae* and *P. niloticum* (table 1; figure 1). Furthermore, unique combinations of character states were also found for both genera of this family (table S3). For extracting character-based DNA barcodes for single populations, the relevant nodes within the species cluster in the NJ tree (figure 1) were selected by means of the CAOS-group file and pure CAs for the groups descending from these nodes were extracted from the ‘diagView_attributes’ file. Overall barcodes with at least three diagnostic characters were detected for eight geographical clusters within the three species (table 2). The character-based DNA barcodes for these entities are composed of species-specific character states and diagnostic characters for the respective populations.

In detail, for two populations of *P. kersteni*, in Namibia (Baynes Mt. and Ongongo), no CAs were found. However, both populations are easily distinguishable from the remaining six populations in Namibia, Kenya and Tanzania through diagnostic characters at five nucleotide positions (348, 381, 429, 466 and 468). The remaining six populations constitute a single entity and could not be discriminated. In contrast, for *P. bicoerulans*, we found character-based DNA barcodes for all four populations from Kenya and Tanzania with diagnostic characters at various nucleotide positions. The two Kenyan populations from Mt Elgon and Mt Kenya can be distinguished by pure CAs at three positions (168, 258 and 433) of the *ND1* fragment. For the third Kenyan population from the Aberdare Mt. and the Tanzanian population from Kilimanjaro, the high number of 15 pure CAs was detected.

Within *P. massaicum*, one population from Kenya (Pm72/Kiboko River) could be distinguished from the five remaining populations in Kenya and Namibia through pure CAs at three nucleotide positions of the *ND1* gene fragment (264, 303 and 460).

## 4. Discussion

Analyses of 833 *ND1* sequences from a wide spectrum of odonate populations, species and genera suggest that character-based DNA barcoding is well suited to the identification of genetic entities at different taxonomic levels. We demonstrate here the potential of the character-based approach and introduce a new marker to common problems in establishing diagnostic barcodes at different taxonomic ranks. The dataset includes populations, species and genera which differ highly in their intra- and interspecific genetic distances. We took advantage of the geographical regions of South and East Africa with their distinct biogeographical and ecological features ranging from tropical, montane and coastal rainforests to desert areas.

By means of the CAOS algorithm, we were able to identify unique combinations of diagnostic characters for most of the pre-described species. The application of single nucleotide characters as barcodes and the identification of population and genus-specific barcodes are a novel addition to the existing barcoding initiatives. We believe that the findings of this study deserve particular attention with respect to three basic questions: How efficient and reliable is the CAOS DNA barcode technique? Why did this study fail to identify diagnostic barcodes for all species included? How useful are DNA diagnostics that can be found for genetic entities at the population level with respect to the discovery of new species?

CAOS is a general framework for character-based DNA barcoding that rapidly identifies CAs for *a priori* defined groups at different taxonomic levels at all nodes of a given phylogenetic tree, even for very large datasets. The Perl scripts developed for this study substantially enhance speed and ease of the data analyses by means of the P-Gnome application. Given that we searched for single diagnostic nucleotide positions (pure CAs) only, instead of complex combinations of nucleotide positions (‘compound CAs’), and also given that only a single short marker sequence was used, we find the outcome of a very high percentage of diagnostic barcodes at different taxonomic levels quite noteworthy and indicative of the power of CAOS barcoding.

The establishment of reliable character-based DNA barcodes depends on the use of an appropriate genetic marker. The *CO1* region of the mitochondrial genome has been the reference marker of choice in DNA barcoding studies so far (e.g. Hebert *et al*. 2004*a*; Kress *et al*. 2005; Bely & Weisblat 2006; Hajibabaei *et al*. 2006; Smith *et al*. 2006; Witt *et al*. 2006). However, as more data have become available, a number of studies have experienced problems with a distance-based, single locus approach (Vences *et al*. 2005; Gomez *et al*. 2007). A distinct overlap in intra- and interspecific distances can cause difficulties in the definition of thresholds for species identification.

Ideally, an appropriate marker for species barcoding should show a high level of *inter*specific variability (to discriminate also between closely related sister species) and at the same time a lower *intra*specific variability (for accurate assignment of specimens to species). Since *ND1* sequences have been known to be highly informative at different taxonomic levels in dragonflies (Hadrys *et al*. 2006; Dijkstra *et al*. 2007; Groeneveld *et al*. 2007), we here tested its suitability as a character-based barcode marker.

### (a) Species level

A subset of data from the family Coenagrionidae, which included a total of 133 samples belonging to two genera and 14 species, was chosen to evaluate the potential of CAOS for character-based DNA barcoding at different taxonomic levels. For 12 out of the 14 species, unique combinations of character states were found in at least 3 out of the 23 selected nucleotide positions. Since 13 analysed species belong to the genus *Pseudagrion*, our results show that *ND1* is an appropriate marker for DNA barcoding also for closely related odonate species, although no diagnostic characters were found to distinguish the sister taxa *P. acacie* and *P. niloticum*. In this case, the minimal divergence of morphological characters has already fuelled a debate about their species status in the past (Dumont 1978; Dumont & Martens 1984). Our results confirm a close genealogical relationship and suggest a recent separation into two species. Additional markers are needed to identify diagnostic barcodes in order to delimitate *P. acacie* and *P. niloticum*.

In general, the level of confidence in a CA to be fixed increases with the number of individuals analysed. In order to claim that a CA is truly fixed, it would be necessary to analyse every single individual of a species (Wiens & Servedio 2000). Obviously, this will never be possible and no absolute certainty for a given characteristic attribute will ever be achieved but reliability of character-based barcodes increases exponentially with each independent diagnostic CA added.

### (b) Genus level

Although not yet approached in any depth, DNA barcoding in genera could be a powerful expansion for taxonomy and for accelerating biodiversity assessment on our planet (Rubinoff *et al*. 2006). In odonates, for example, identification keys for larvae based on morphological characters often do not exceed the family level and some genera do not form monophyletic groups (Artiss *et al*. 2001). Thus, DNA barcodes at the genus level could facilitate the assignment of unknown samples. We found character-based DNA barcodes for 21 out of 25 genera. For example, at 5 out of the 30 chosen nucleotide positions of the *ND1* gene region, all specimens of the genus *Teinobasis* differed from individuals of the genus *Pseudagrion*, which both belong to the family Coenagrionidae. Only within the Aeshnidae, four out of six genera failed to deliver genus-specific barcodes. Those findings are especially interesting, since Aeshnidae are supposed to be one of the most ancient dragonfly groups and one would expect a clear genetic divergence. However, the family Aeshnidae harbours species which are the most powerful flyers and their high dispersal potential possibly acts against fast genetic divergence.

### (c) Population level

Resolution of unrecognized, cryptic biodiversity patterns is a major task. Character-based DNA barcoding can nicely complement morphological, behavioural and ecological data in the process of species discovery in this important biodiversity assessment endeavour. For example, marine environments present particular challenges to biodiversity assessment where limitations of traditional species identification may grossly underestimate the number of species. Another example is tropical rainforests where a high number of habitat specialists face strong niche conservatism and fragmentation events at the same time. Reliable detection and rapid monitoring of biodiversity patterns could help to identify CUs and accelerate management actions. Subsequently, it would be particularly desirable to also examine whether reliable population (intra-)specific barcodes exist in order to establish new hypotheses of species existence and set benchmarks for future monitoring studies. Such DNA barcode diagnosable units could potentially be raised to species status if corroborating evidence is collected and taxonomic revision accomplished (DeSalle *et al*. 2005; Desalle 2006).

Overall, our dataset shows the potential existence of sympatric (*Trithemis stictica*) as well as allopatric taxonomic entities (e.g. *T. stictica, Coryphagrion grandis, P. kersteni, P. massaicum, P. bicoerulans*), in the context of no, minimal or non-correlating morphological change. In the data subset of the genus *Pseudagrion*, several geographical clusters were identified in all three species. The 10 individuals of *P. kersteni* from the two northern Namibian populations, Baynes Mt and Ongongo, are well separated by five CAs from all other populations. The third Namibian population, Naukluft, is also separated from the former two and all other populations by one diagnostic character. Interestingly, neither the Kenyan nor Tanzanian populations show diagnostic characters yet. In the related species, *P. massaicum*, we found three pure CAs in the *ND1* sequences of all 12 individuals of the Kenyan population (Kiboko River) separating these from the Namibian populations. The remaining six individuals from Kenya, the ‘Pemba River population’, share a character-based DNA barcode with the Namibian populations. The most interesting example is the montane rainforest species *P. bicoerulans*. Here, character-based DNA barcodes could be established for all geographical locations. Interestingly, the barcodes neither coincide with the different colour patterns nor with spatial separation of the populations (Hadrys *et al*. 2006). While the two Kenyan populations from Mt Elgon and Mt Kenya can be reliably distinguished by three pure CAs, an unexpected high number of 14 CAs were detected for the remaining two populations from Kenya and Tanzania (Aberdare Mt and Kilimanjaro). The data suggest that these populations have been isolated for long periods of time and that speciation processes are either in progress or incipient.

The results, based on a relatively short sequence marker, show that character-based DNA barcoding with CAOS can be an effective and reliable means for identifying diagnostics for populations and CUs. The use of the barcode approach in this way could nicely complement organismal data in order to unravel speciation processes and identify cryptic species (DeSalle *et al*. 2005; Desalle 2006). We emphasize, however, that the DNA barcodes in and of themselves do not establish that these potential units are indeed new species. Integrated taxonomic approaches (Rubinoff 2006*a*,*b*) are required to accomplish this species discovery process.

## 5. Conclusions

Establishing DNA barcodes to identify previously defined groups of organisms at any taxonomic level is a powerful application of modern technology to biodiversity study. In this study, we achieved the first step for character-based DNA barcoding by highlighting the principal potential and effectiveness for identifying diagnostic DNA barcodes at different taxonomic levels. Those character-based DNA barcodes are directly applicable to high-throughput analyses and therefore are a powerful tool when applied to large-scale and long-term biodiversity assessment studies. Not being distance based, these barcodes can be readily incorporated into a concatenated data matrix which contains further information in form of characters.

When establishing a barcode, the most critical parameters are sample size and the number of CAs. While the addition of genetic markers is only a matter of routine work to be done, sample size can be critical when it comes to endangered species or species of specific environments that are not easy to encounter. In dragonflies, for example, rainforests and arid areas harbour a high number of rare species, which often are poorly known and occur in small population sizes threatened by habitat fragmentation (e.g. Hadrys *et al*. 2006; Paulson 2006). Although CAs found in a single individual may possibly not be representative for all members of this species, the DNA barcode obtained for a single specimen can still be useful in the overall process of species identification. Simply put, a diagnostic species barcode derived from several specimens is obviously a more reliable identifier than a DNA barcode determined from a single specimen. However, the inclusion of a single specimen barcode can provide an important benchmark for this species within the group of interest. Besides, overall reliability of a barcode increases exponentially with each additional CA, even though only a single specimen is analysed.

Given the different levels, problems and the characteristics of the group we have approached, the success we report here by using only the simple character-based DNA barcodes (sPu and sPr (≥80%) CAs) seems even more promising. We conclude that the character-based barcode approach can offer an efficient and reliable method for accurate species and conservation unit identification, with the great advantage of being compatible with traditional taxonomy in a wider context.

## Figures and Tables

**Figure 1:**
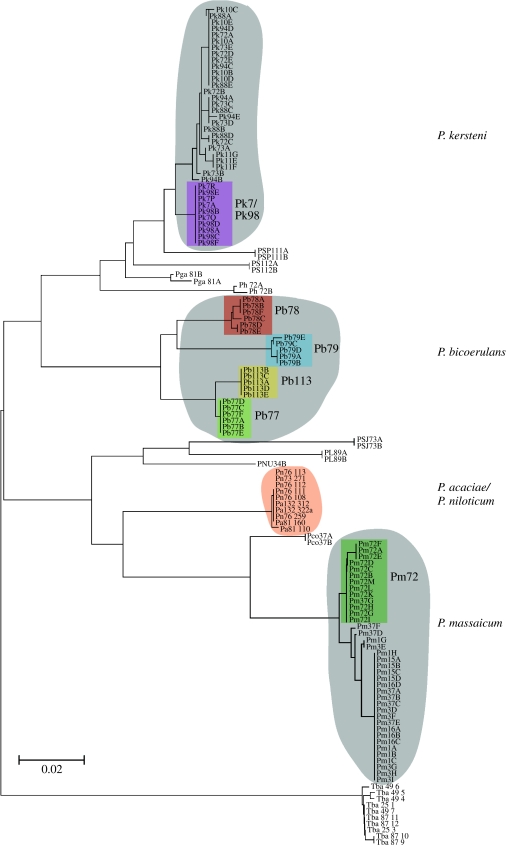
Neighbour-joining tree based on K2P distances for 14 odonate species of the family Coenagrionidae; this subset is used for demonstrating the power of the character-based DNA barcoding technique at the population level; clades framed in grey include species for which at least four populations are analysed; the clade framed in red indicate a shared character-based DNA barcode by two species; coloured squares within grey frames highlight geographical clusters within species for which specific character-based DNA barcodes are identified (for more details see text).

**Table 1:** Character-based DNA barcodes for 14 odonate species belonging to the family Coenagrionidae; (character-based DNA barcodes of all 64 analysed species are shown in table 2 of electronic supplementary material); Character states (nucleotides) at 23 selected positions of the *ND1* gene region (ranging from position 201–444); taxa, abbreviations according to table S1*a*,*b*; numbers of individuals analysed per species are given in brackets; Grey cells indicate the occurrence of three or all four nucleotides at this particular position in the sequences of samples of the corresponding species; When two character states were present at a given position the number of individuals showing each character is given in brackets; taxa abbreviations in bold indicate shared character-based DNA barcodes for two species; taxa abbreviations in red indicate that three or fewer diagnostic characters were found between the two adjacent taxa.

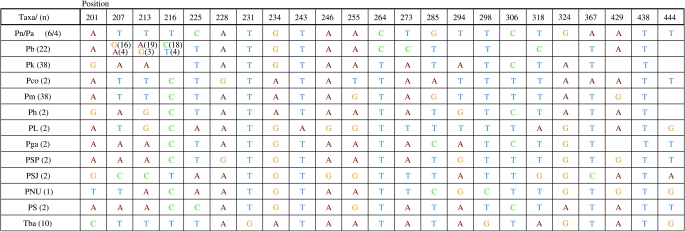

**Table 2:** Example of character-based DNA barcodes; unique combinations of character states for individual populations of *Pseudagrion kersteni* (Pk), *P. bicoerulans* (Pb) and *P. massaicum* (Pm); the character-based DNA barcodes include character states for species identification (table S3) and additionally character states that are diagnostic for populations; the populations are identified by &lsquo;locality codes&rsquo; (PopLc), for example, Pk98=Baynes Mt., Namibia (*a*); the two lines showing only species abbreviation without locality code, Pm (25) and Pk (28), include the remaining populations of these species that share a character-based DNA barcode. Details of populations (locality, name, country, longitude/latitude and abbreviation used) are given in table S4 (electronic supplementary material)

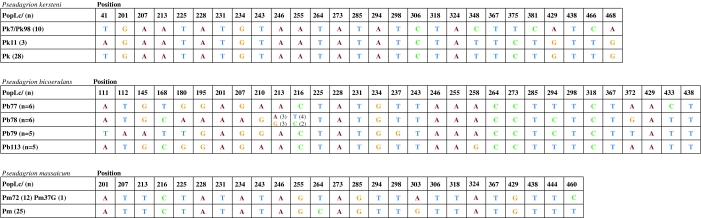

## Supplementary Material

Guide tree (NJ tree) for CAOS analysis Neighbour-joining tree based on Kimura-2-parameter distances of ND1 sequences of 833 specimens belonging to 64 Odonate species; Guide tree for character-based DNA barcoding by means of the characteristic attributes organisation system (CAOS) algorithm
