# Supplementary material for: Character-based DNA barcoding allows discrimination of genera, species and populations in Odonata
Source: Proc Biol Sci. 2007 Nov 8;275(1632):237–47. doi: 10.1098/rspb.2007.1290 (PMC2212734; doi:10.1098/rspb.2007.1290)
Supplement: Supplementary file 1 [file rspb20071290s01.pdf]

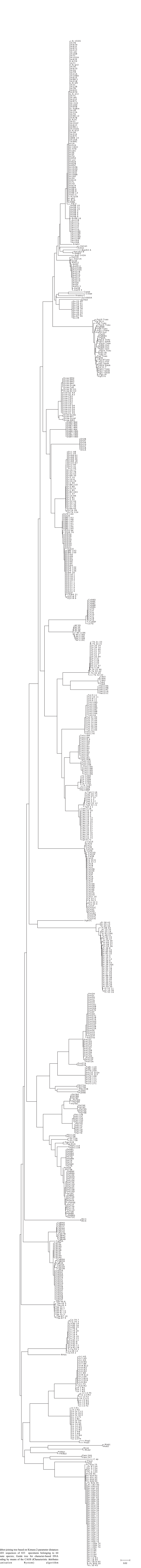

Neighbor-joining tree based on Kimura-2-parameter distances of ND1 sequences of 833 specimens belonging to 64 Odonate species; Guide tree for character-based DNA barcoding by means of the CAOS (**C**haracteristic **A**ttributes **O**rganisation **S**ystem) algorithm
