# Supplementary material for: Character-based DNA barcoding allows discrimination of genera, species and populations in Odonata
Source: Proc Biol Sci. 2007 Nov 8;275(1632):237–47. doi: 10.1098/rspb.2007.1290 (PMC2212734; doi:10.1098/rspb.2007.1290)
Supplement: Supplementary file 2 [file rspb20071290s02.doc]

**ESM-Table 1*a/b*.** Species of the suborders Anisoptera (*a*) and Zygoptera (*b*) included in this study; Ind. = number of individuals analysed; ID = abbreviation of species name; Loc. = number of sample sites (localities).

**ESM-Table 2**: Character-based DNA barcodes for 64 odonate species; Character states (nucleotides) at 23 selected positions of the ND1 gene region (ranging from position 201 – 444); Taxa = abbreviations according to Tables 1a and 1b; numbers of individuals analysed per species are given in brackets; Grey cells indicate the occurrence of three or all four nucleotides at this particular position in the sequences of samples of the corresponding species; When two character states were present at a given position the number of individuals showing each character is given in brackets; taxa abbreviations in bold indicate shared character-based DNA barcodes for two species; taxa abbreviations in red indicate ≤ three diagnostic characters were found between the two adjacent taxa.

**ESM-Table 3**: Character-based DNA barcodes at the genus level: Character states (nucleotides) at 30 selected positions of the ND1 gene region (ranging from 25 – 434); dashed cells indicate the occurrence of three or all four bases at this particular nucleotide position within a genus; cells were shaded grey if two different bases were present within a genus; numbers of analysed species and individuals are shown in brackets; Genus names in red indicate ≤ two diagnostic characters were found.

**ESM-Table 4**: Populations of three *Pseudagrion* species, which can be identified by character-based DNA barcodes; for each population the name of the sample site (locality), country, abbreviation, number of individuals analysed and coordinates of the locality are given.
